# The Role of Protein and Lipid Clustering in Lymphocyte Activation

**DOI:** 10.3389/fimmu.2021.600961

**Published:** 2021-03-09

**Authors:** Rachel E. Lamerton, Abbey Lightfoot, Daniel J. Nieves, Dylan M. Owen

**Affiliations:** Institute of Immunology and Immunotherapy, School of Mathematics and Centre of Membrane Proteins and Receptors (COMPARE), University of Birmingham, Birmingham, United Kingdom

**Keywords:** nano-clustering, lymphocytes, T cell synapse, B cell synapse, lipid rafts

## Abstract

Lymphocytes must strike a delicate balance between activating in response to signals from potentially pathogenic organisms and avoiding activation from stimuli emanating from the body's own cells. For cells, such as T or B cells, maximizing the efficiency and fidelity, whilst minimizing the crosstalk, of complex signaling pathways is crucial. One way of achieving this control is by carefully orchestrating the spatiotemporal organization of signaling molecules, thereby regulating the rates of protein-protein interactions. This is particularly true at the plasma membrane where proximal signaling events take place and the phenomenon of protein microclustering has been extensively observed and characterized. This review will focus on what is known about the heterogeneous distribution of proteins and lipids at the cell surface, illustrating how such distributions can influence signaling in health and disease. We particularly focus on nanoscale molecular organization, which has recently become accessible for study through advances in microscope technology and analysis methodology.

## Introduction

In order for the immune system to effectively neutralize pathogens, cells must take part in complex cell-cell communication interactions, such as those occurring between T or B cells and antigen presenting cells (APCs), such as dendritic cells. At the level of whole cells, these interactions take place through the formation of the immunological synapse (IS). Rather than being uniformly distributed over the IS, proteins are segregated into a bullseye-like configuration, made up of three concentric sections (1) easily resolved by conventional fluorescence microscopy. Termed supramolecular activation clusters (SMACs), the original findings placed T cell receptor (TCR) complexes in the central SMAC (cSMAC), adhesion molecules, such as the integrin LFA-1 in a narrow ring-shaped peripheral SMAC (pSMAC) and negative regulators, such as the large phosphatase CD45, with a dense cortical actin meshwork in the distal SMAC (dSMAC). Since then, it has become widely accepted that actively signaling TCR molecules are more likely to be found in the distal regions of the synapse and subsequently migrate into the cSMAC (2–4).

Whilst macroscale organization is also observed in B and NK cells, they form their own uniquely structured IS for their specific functions. The B cell synapse typically lacks a well-defined dSMAC, with B cell receptors (BCRs) concentrated in a large cSMAC specialized for gathering antigen for internalization and further intracellular processing (5). The NK cell synapse is more complex, with differently structured synapses corresponding to different cellular outcomes—the lytic synapse, the inhibitory synapse and the regulatory synapse (6).

The lytic synapse is somewhat similar to the B cell arrangement, with a cSMAC containing lytic granules and the microtubule organizing center (MTOC) with adhesion molecules located in the pSMAC. The inhibitory IS differs, with killer immunoglobulin-like receptors (KIRs) gathering in the cSMAC. In each type of synapse, on top of these cell-scale organizational layers on the scale of microns, is nanoscale molecular organization—the clustering of proteins and lipids. The composition and properties of the membrane, therefore, have a profound effect on cell activation. These are reviewed in (7).

**Figure 1:**
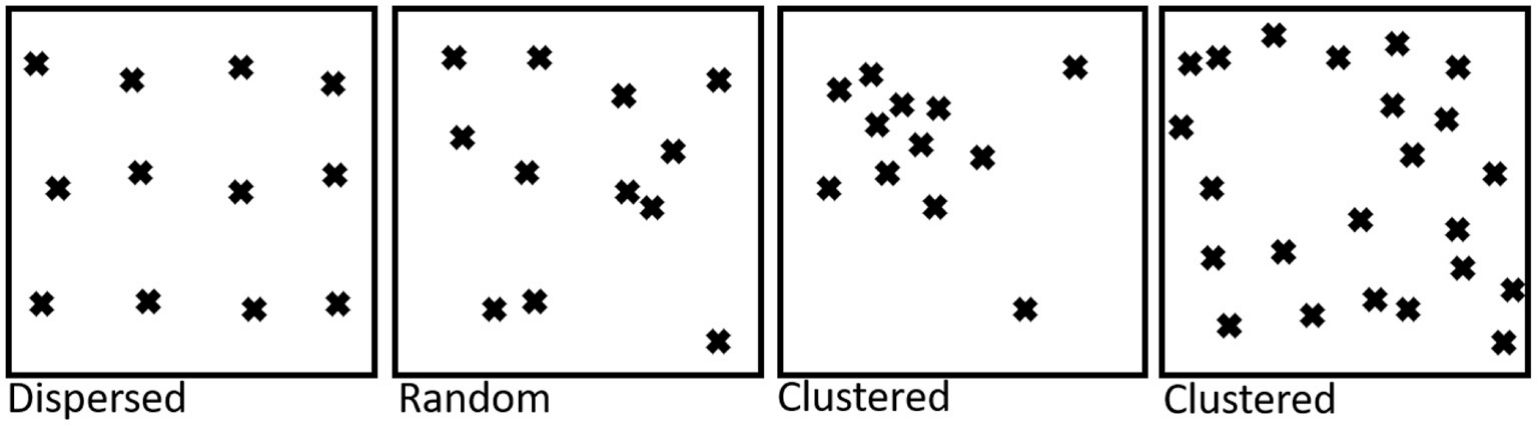
Illustration of different molecule (protein or lipid) distributions on the cell surface. For dispersed distributions, the average distance between molecules is larger than would be expected for randomly distributed molecules. In both the clustered examples shown here, the average nearest neighbor distance is smaller than expected for randomly arranged molecules.

There are a number of ways of mathematically defining what we mean when we use the term “molecular clustering” (Figure 1). One of the simplest is that on average, the distance from one molecule, for example LAT, to its closest LAT neighbor is shorter than would be expected had all the LAT molecules been randomly distributed. Furthermore, the difference should be large enough that with a given statistical test and experimental power, we can distinguish the two cases, given the random degree of clustering that might occur in any control dataset.

The same concept can be applied to lipids—if the average distance from a sphingomyelin molecule to its nearest neighbor is smaller than had all the sphingomyelin lipids been randomly distributed, and by enough that this can be confirmed with statistical significance, then sphingomyelin is said to be clustered. In most biological scenarios almost all non-random distributions of molecules will probably result in clustering but not necessarily to a degree that chance clustering in a random distribution can be ruled out with a given statistical certainty.

Almost all descriptions of heterogeneous molecular distributions (proteins or lipids) are therefore descriptions of clustering. It should also be made clear that protein clustering does not imply any dynamic process or change in distribution, which would be correctly described as an increase or decrease in clustering. Here, we use clustering to denote a state, not a process.

## Protein Clustering

In T cells, many of the key proximal signaling molecules; TCRs (8, 9), LAT (10, 11), Lck (12), and ZAP-70 (13, 14) have been shown to form nanoclusters. With the need for both sensitivity and selectivity in T cell responses, the spatial organization of TCRs is emerging as a key factor in appropriate and adequate T cell signaling. TCRs are generally thought to pre-cluster on resting cells with several studies detecting clusters using optical microscopy methods (8, 10), with approximately 7–30 TCRs per cluster, and an average radius of 35–70 nm (10). Upon cell activation, clusters become increasingly dense, with denser clusters being linked to phosphorylation, and higher signaling efficiency. Interestingly, cluster density appears to be determined by the dose and affinity of the MHC-antigen to the TCR, suggesting a relationship between antigen and signaling mediated through clustering (8).

Similar to T cells, studies have shown resting BCRs in IgM and IgG producing B cells cells are clustered with an average radius below 60 nm, though a much broader range of cluster sizes is observed in resting IgG producing cells (15). These clusters might be lipid dependent and exclude the phosphatase CD45 (16). In contrast to T cells, the density of clusters in B cells tends to decrease upon activation (15).

In NK cells, the nanoclustering properties differ again. Rather than relying on one dominant activating receptor being triggered, as occurs in T and B cells, the outcome of NK cell interactions is controlled by the balance of activating and inhibitory signals from multiple receptors. KIR2DL1 is an inhibitory receptor arranged in clusters on resting NK cells (17). Upon exposure to the activating receptor NKG2D, causing formation of the cytolytic immune synapse, the KIR2DL1 clusters become smaller and denser. Resting KIR2DL1 clusters, as measured on IgG1-coated control coverslips, were approximately 122 nm in diameter, decreasing in size by almost 20% in samples activated by the NKG2D receptor. There was also a 58% increase in KIR2DL1 cluster density between the IgG1 control and NKG2D receptor coverslips (17).

One of the key areas of debate is now whether important signaling molecules are pre-clustered on the surface of resting cells and if so, whether those pre-stimulus clusters are essential. In 2005, a FRET study suggested BCRs existed as monomers on resting cells (18), a finding that has also been shown by diffusion studies (19). More recent studies have shown pre-existing BCR clusters in resting cells using electron microscopy (20) and single molecule localization microscopy (SMLM) techniques, such as direct stochastic optical reconstruction microscopy (dSTORM) and photoactivated localization microscopy (PALM) (15). Rossboth et al. (21) suggest that clusters may be detected due to overcounting errors inherent to SMLM techniques. They carried out robust studies using dSTORM, PALM, and stimulated emission depletion (STED) to show that TCRs are distributed randomly across the membrane in resting cells. In a separate study, the notion that pre-clustering plays a role in antigen sensitivity and specificity was questioned. Using FRET and a single-molecule fluorescence brightness analysis method, monomeric TCR-CD3 complexes were found to initiate intracellular signaling, rather than TCR-CD3 oligomers (22).

Overall, much uncertainty about the presence or role of pre-clusters still exists. The fact that pre-clustering has been detected by independent techniques (SMLM, FRET, EM etc), and in different cell types suggests non-random distributions on the surface of non-stimulated cells is likely, at least for some molecules. As we discussed in the definition of clustering, different tests will have different statistical power and may have been testing different definitions of clustering. If clustering is low level with only small deviations from random distributions, these different tests could account for the discrepancies and controversy. Going forward, the community might agree a definition for molecular clustering and work to establish how clustering, by that definition, would be manifested in different data types. In parallel, work is needed on the experimental sample system to agree clear definitions of “resting cells” and other biological descriptors to foster consistency between systems used in competing claims.

## Lipid Clustering

The original “lipid raft hypothesis” suggested that interactions between cholesterol and lipids with highly saturated acyl chains can cause cholesterol clustering in which cholesterol-proximal lipid acyl tails become highly ordered, as opposed to the disordered tails in the surrounding membrane. These clusters, it was hypothesized, can also preferentially accommodate some proteins whilst excluding others (23), therefore leading to clustered distributions for both the “raft enriched” and excluded proteins (Figure 1). There is a wide variety of nomenclature in the literature for describing the lateral distribution of lipids—rafts, domains, territories, islands, and some open questions about whether lipids “cluster” in the same way as has been observed for proteins. Mathematically, however, the lipid raft hypothesis, ordered phase domains and other similar models are describing clustering, even if the particular lipid distribution involves areas of exclusion (Figure 1). If you were to take a particular lipid species, e.g., palmitoyl sphingomyelin (PSM) for example, and plot the coordinates of each molecule on the cell surface, almost all these membrane models would result in clustering—the distance to a nearest neighbor would be smaller than would be expected for a completely random distribution of the lipid in question. We therefore consider rafts, domains as well as concepts such as islands and territories—any area in which a specific type of lipid is “enriched” or “excluded” to be a form of clustering (which may or may not be detectable, depending on the statistical strength of the experiment and analysis). However, the exact properties of the clustering, the biophysical determinants and the functions of lipid clusters could be very diverse from protein clusters.

One method for studying such distributions is using detergents, where, under specific conditions, clusters of sterols and lipids with ordered acyl tails are resistant to solubilisation. Detergent solubilisation assays of T cell derived giant plasma-membrane vesicles (GPMVs), report that non-activated TCRs are absent from detergent-resistant fractions and that TCRs only translocate into detergent-insoluble fractions (i.e., co-cluster with ordered-tail lipids and sterols) upon receptor activation (24). In contrast, the adaptor protein LAT, the Src-family kinase Lck, and the CD4/CD8 co-receptors involved in TCR signaling can be recovered in detergent-resistant fractions independently of TCR triggering (25, 26). Similarly, in B cells, the BCR is not present in detergent-resistant fractions prior to crosslinking, and will translocate to detergent-resistant fractions which are also enriched with the Src-family kinase Lyn following BCR ligand binding (27). The discovery that signaling complexes co-cluster with sterols and ordered acyl tail lipids which are resistant to detergent solubilisation, following receptor activation suggests a functional role of lipid clusters as hotspots that compartmentalize and regulate immunoreceptor activation and subsequent downstream signaling events (26). The use of detergent resistance, however, has started to fall out of favor, mainly due to the potential artifacts of the procedure (for example due to using cold temperatures) and often contradictory results, reviewed in Lichtenberg et al. (28). Most studies now focus on the less invasive optical microscopy methods, reviewed in Sezgin et al. (29).

Environmentally-sensitive lipid dyes such as Laurdan and Di-4-ANEPPDHQ exhibit spectral shifts in their fluorescence emission dependent on the degree of lipid packing in the membrane. These probes are solvatochromic, sensing the polarity of their local solvent, and, in the case of membranes therefore, the relative penetration of polar water molecules into the bilayer. Multispectral imaging can therefore be used to probe and quantify membrane lipid packing, which is dependent on the ordering of the lipid tails by sterols (30). Inconsistent with detergent solubilisation assays, Laurdan labeling of cells shows that TCR proteins in resting cells co-localize with areas of dense lipid packing and aggregate into even larger TCR clusters following cross-linking (31). The disparity between results observed using microscopy and those found using detergent-resistant solubilisation assays might arise from the instability of dynamic nanoclusters or artifacts from the detergent solubilisation process, in particular when membrane constituents might be associated with cortical actin.

Probes such as these can be used to map membrane properties across the immunological synapse. Interestingly, condensation of the membrane surrounding the TCR complex at the T cell immunological synapse has been observed (32, 33). The pattern of increased membrane ordering at the immunological synapse periphery supports the proposed pattern of actively signaling TCR microclusters within the SMACs (34). Furthermore, application of small molecule agents such as the cholesterol analog 7-ketocholesterol (7KC), which disrupts lipid ordering, also disrupts T cell activation and synapse formation (35). Methyl-β-cyclodextrin depletion of cholesterol in the plasma membrane has been shown to both inhibit and enhance the activation of T cells, depending on experimental conditions (36, 37). It was recently found that following cell treatment with the naturally occurring analog cholesterol sulfate, TCR nanoclusters were disrupted, leading to reduced avidity for peptide-MHC and reduced CD3 ITAM phosphorylation (38). This points the way toward potential manipulation of membrane lipids in order to control molecular distributions (clustering) and function and, therefore, lymphocyte signaling.

Another way membrane lipids can influence the distribution and dynamics of membrane proteins is via sub-synaptic vesicles. Using SMLM and total internal reflection fluorescence microscopy (TIRF), LAT-containing sub-synaptic vesicles were found to be recruited to the plasma membrane in early cell activation, and it was found that plasma-membrane associated LAT may not be involved in signaling (11). Since then, it is believed that two phases of T cell activation occurs, initiated first by pre-existing clusters on the plasma membrane and prolonged by the recruitment of LAT containing vesicles that were found to have higher membrane order than non-LAT containing vesicles (39, 40). Lipid composition, therefore, might represent a novel mechanism for organizing cargo transport by intracellular vesicles.

Finally, in addition to regulating protein distributions and function, high lipid tail order has also been proposed to play a protective role for cytotoxic CD8+ T cells, protecting against accidental death by repelling perforin (41). In these cells, phosphatidylserine is enriched at the immunological synapse following antigen recognition (42). The negative charge of phosphatidylserine was found to inactivate residual perforin as an additional mechanism to prevent accidental cell death and allow for successive killing of target cells (41). With this in mind, elevated levels of negatively charged lipids on virus envelopes and cancerous cell membranes may be immunoevasive.

## Protein and Lipid Clustering Relevant to Disease

Understanding of lymphocyte signaling protein cluster properties, and their regulation, has significantly advanced over the last 20 years, however, the next challenge is to exploit this knowledge in the context of health and disease. There is currently a lack of knowledge as to how these clustering properties relate to disease states and whether their exploitation could improve current clinical treatments.

One study looking at diffuse large B-cell lymphomas (DLBCLs) has shown that, of the 5 activated B-cell-like DLBCL cell-lines studied, all had pronounced BCR clustering, a phenomenon that was not observed in the 16 other cell lines tested from a variety of different B cell cancer types (43). Furthermore, decreased BCR diffusion in these lines was also observed, and it was suggested these clusters were actively signaling, with phosphotyrosine accumulation in these cells. This so-called “chronic active” signaling was required for cell survival, therefore, highlighting a role for the BCR clusters and the signaling they induce in activated B-cell-like DLBCL, and revealing a possible new treatment target.

Inappropriate clustering has also been linked to autoimmune diseases. The R620W variant in protein tyrosine phosphatase non-receptor type 22 (PTPN22) is associated with rheumatoid arthritis, lupus and type one diabetes and is a mutation present in the human population (44). More recently, Burn et al. (45) showed that PTPN22 inhibits LFA-1 signaling, with the R620W variant being loss-of-function. It was found that the mutation, in a non-catalytic protein binding domain, alters the clustering profile of PTPN22 with a resulting failure to de-cluster upon cell stimulation. The R620W mutation was also shown to be associated with changes in LFA-1 integrin clustering in migrating T cells (46).

Lipid clustering has also been implicated in a number of lymphocyte-related diseases, including cancer and autoimmunity. Hallmarks of systemic lupus erythematosus (SLE) include chronic immune cell activation and increased serum lipids, with interplay between the two being demonstrated (47, 48). High membrane order and increased cholesterol and glycosphingolipid levels have been observed in the plasma membrane of T cells from SLE patients (49, 50). The altered lipid environment is believed to drive an increase in glycosphingolipid expression in CD4+ T cells in a liver X receptor (LXR) dependent manner.

*In vitro* studies suggest that T cell function in SLE patient derived cells can be restored through normalization of glycosphingolipid expression using LXR antagonists (51). Additionally, LXR activation of CD4+ T cells from healthy donors can also upregulate expression of glycosphingolipids leading to reduced membrane order at the TCR immunological synapse. Altered spatiotemporal distribution of lipids promoted Lck recruitment to the immunological synapse and increased phosphorylation of CD3 and LAT, dysregulating effector functions via disruption of ordered lipids (52). Additionally, kinase phosphorylation in SLE patient-derived T cells is restored by statin inhibition of cholesterol synthesis (53). The cytotoxic effects of CD8+ T cells can be heightened by ablation or inhibition of cholesterol esterification enzyme ACAT1, which is upregulated upon TCR activation (54). Together these findings suggest that tight regulation of lipid properties and distributions is necessary for normal T cell function. There are numerous processes that result in diseases associated with lymphocytes where cholesterol plays a role. For example HIV entry, associated with the surface marker CD4, is significantly impaired by cholesterol depletion from the plasma membrane (55). Further, although we have focussed on cholesterol here, many other lipids display clustering and for a more complete review, we point the reader to Wu et al. (56). Many of these are also involved in regulating membrane order and, therefore might be relevant to human health and disease (57) including cancer and autoimmune disease.

## Conclusions

In lymphocytes, protein clustering has been well-documented on the micron-scale and with the development of super-resolution microscopy, is beginning to be characterized on the nanoscale. The challenge remains as to what biophysical mechanisms are governing nanocluster formation and what the ultimate function of such protein distributions is.

Cluster formation is likely due to a variety of competing and non-exclusionary phenomena. Prominent among these are the clustering properties of the lipids themselves within the plasma membrane. This is important as it brings into play a number of small molecule pharmacological agents, and even genetic manipulations, to control the lipidome and, therefore, cell fate and function. Protein-protein interactions and corralling by the cortical actin meshwork are also potentially important mechanisms and, thus, therapeutic targets.

The function of protein clustering in T cells has only been minimally explored, in part due to the lack of molecular tools to selectively manipulate the systems. In other protein pathways clustering has been shown to create digital switches, for example, in Ras signaling (58). Signaling via two protein species is dependent on the frequency of collisions between them as they diffuse, say in a 2D membrane. A similar situation exists for example in the rate of a chemical reaction as two substrates diffuse and react to form a product. In reaction kinetics, whether a substrate is dimeric or oligomeric and whether there is cooperativity between sites (allostery) will change the rate of product production. In the same way, clustering will change the rate of protein-protein interactions in a signaling interaction (59).

**Figure 2:**
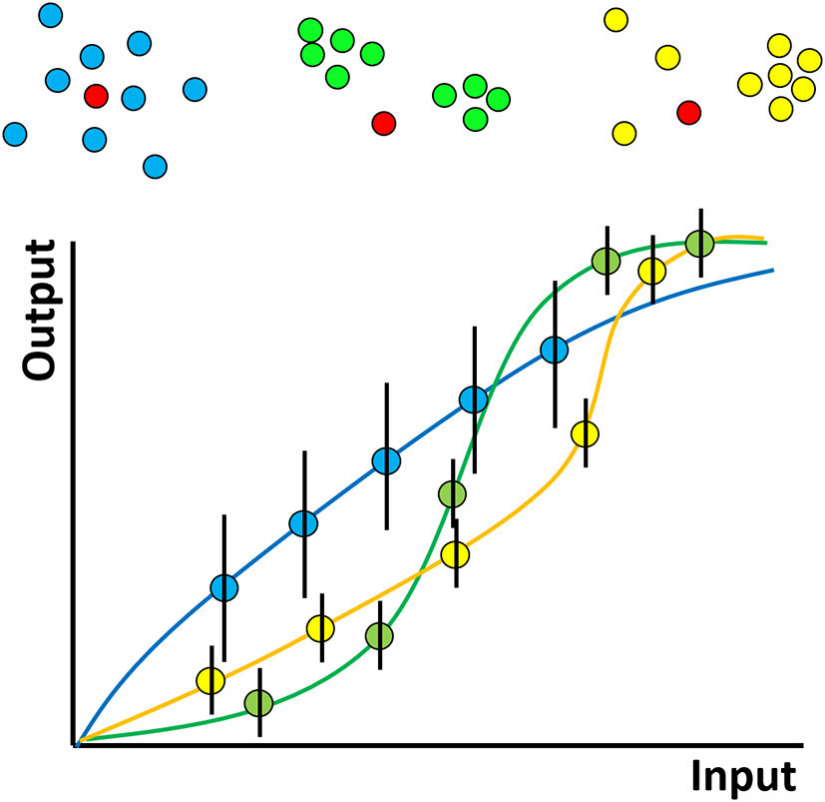
Hypothesized effect of molecular nanoclustering on input-output relationship of a generalized signaling pathway for two proteins. A hypothetical kinase for example (red) can diffuse in the membrane and phosphorylate a substrate with different clustering properties (blue, green, yellow). The input can be conceptualized simply—as the amount of active kinase or more generally—say as the strength of antigen binding by a receptor. The output might be proximal downstream phosphorylation levels more distal, such as gene expression—analogous to the rate of production of product in a chemical reaction. The shape of the curve relating these two depends on the stoichiometry, oligermerisation and cooperativity of the molecules involved, just like a classical chemical reaction. In a cell signaling pathway however the situation will be complex, with many interacting partners, feedback and so on. The exact shapes of such hypothetical curves therefore remain an area for future study.

The hypothesized role of clustering can be illustrated by considering a hypothetical experiment in which two types of interacting molecules–say kinases and their substrates (which may be proteins or lipids, such as inositides) are observed (Figure 2). The “input” to the system represents the amount of kinase activity e.g., it may represent kinase concentration, activation status or elapsed time through the experiment. The “output” here might represent the amount of phosphorylated substrate. We hypothesize that clustering determines the shape of the curve relating this input and output and in particular, that clustering produces more digital relationships featuring input thresholds required to achieve any significant output. On top of this, phosphorylation events are stochastic (because of the random nature of diffusion) and so if the experiment were repeated many times, different amounts of phosphorylation would be observed each time, for a given input. In Figure 2, this is represented by the hypothesised size of the error bars. We further hypothesize that clustering affects the size of these variances, decreasing “noise” in the system.

In reality of course, a cellular signaling pathway will have many members and might involve complex feedback processes. In addition, while many studies have been performed on proteins, here, lipids play a dual role. They may represent members of a signaling pathway themselves (e.g., phosphatidylinositol) and therefore their distribution/clustering may be directly important, or they may be indirectly involved by influencing the clustering of proteins. Despite the complexity of a complete signaling pathway, there will still be some input-output curve for the entire pathway. In lymphocytes, for example, the input might represent the number or strength of engaged TCRs/BCRs and the output might be the level of downstream phosphorylation or even gene expression. We hypothesize that even in this complex case, the shape of the curve will be influenced by the clustering properties of the many proteins and lipids that constitute the pathway. Since many pathologies, for example autoimmune disease, may be caused by modified immune cell activation thresholds, understanding molecular clustering, and how to manipulate it, may lead to new types of therapeutic interventions.

Clearly, several open questions remain to be answered. For example, to relate protein clustering to the concepts of allostery, stoichiometry and competition in classic enzyme kinetics, requires accurate measurements of protein oligomers even down to the level of dimers. In some contexts, this is possible, but precise molecular counting and localization on the scale of proteins is still difficult. Since much of the recent progress has been made using optical microscopy, Table 1 summarizes the main methods that have been applied to study protein and lipid clustering in lymphocytes and, in our opinion, what developments are needed to further push the boundaries of our understanding.

**Table 1:** Fluorescence methods applied to study membrane protein and lipid clustering, their limitations and suggestions for future development.

**Method**	**Limitations**	**Required developments**
Fluorescence microscopy with environmentally sensitive probes	Dyes change the composition of the bilayers and may alter properties (60). They also do not generally allow the environment around specific molecules to be probed as the dyes are usually untargeted. Probes often have low brightness and photostability.	Targeted, leaflet specific probes compatible with super-resolution microscopy (61–63).
Single molecule localization microscopy (SMLM)	Overcounting due to multiple blinking makes quantifying small clusters challenging (64, 65). Labeling methodology generally limited to proteins.	New, smaller probes, with well-characterized photophysics and new analysis methods are needed. Further, the technology for labeling lipids—to allow lipid clustering to be analyzed in the same framework as proteins is also lacking and developments in this area would be a significant boon.
Diffusion measurements such as fluorescence correlation spectroscopy (FCS)	Generally, point measurements are used making mapping heterogeneity over the cell challenging. Can be difficult to interpret data in complex membrane geometries.	Area based FCS measurements such as Imaging FCS (66). Greater computational modeling approaches to single-molecule tracking data.
Whole cell imaging	Generally, artificial synapses are studied but more physiological insights could be derived from cell-cell conjugate systems (67).	Application of light-sheet based approaches for long term, 3D imaging.

In addition to microscopy, tools to specifically alter protein and lipid nanoscale clustering are required in order to better draw causal links with cell phenotypic outcomes. On the biological side, the role of pre-clustered surface proteins is an emerging topic, still controversial but has the potential to have a significant impact on our understanding of how B and T cells are regulated.

## Author Contributions

RL, AL, DN, and DO wrote the manuscript. All authors contributed to the article and approved the submitted version.

## Conflict of Interest

The authors declare that the research was conducted in the absence of any commercial or financial relationships that could be construed as a potential conflict of interest.

## References

[1] Monks CR Freiberg BA Kupfer H Sciaky N Kupfer A. Three-dimensional segregation of supramolecular activation clusters in T cells. Nature. (1998) 395:82–6. 10.1038/25764 9738502

[2] Bunnell SC Hong DI Kardon JR Yamazaki T McGlade CJ Barr VA. T cell receptor ligation induces the formation of dynamically regulated signaling assemblies. J Cell Biol. (2002) 158:1263–75. 10.1083/jcb.200203043 12356870 PMC2173229

[3] DeMond AL Mossman KD Starr T Dustin ML Groves JT. T cell receptor microcluster transport through molecular mazes reveals mechanism of translocation. Biophys J. (2008) 94:3286–92. 10.1529/biophysj.107.119099 18199675 PMC2275686

[4] Soares H Henriques R Sachse M Ventimiglia L Alonso MA Zimmer C. Regulated vesicle fusion generates signaling nanoterritories that control T cell activation at the immunological synapse. J Exp Med. (2013) 210:2415–33. 10.1084/jem.20130150 24101378 PMC3804939

[5] Batista FD Iber D Neuberger MS. B cells acquire antigen from target cells after synapse formation. Nature. (2001) 411:489–94. 10.1038/35078099 11373683

[6] Mace EM Orange JS. Multiple distinct NK-cell synapses. Blood. (2011) 118:6475–6. 10.1182/blood-2011-10-381392 22174302

[7] Pettmann J Santos AM Dushek O Davis SJ. Membrane Ultrastructure and T Cell Activation. Front Immunol. (2018) 9:2152. 10.3389/fimmu.2018.02152 30319617 PMC6167458

[8] Pageon SV Tabarin T Yamamoto Y Ma YQ Bridgeman JS Cohnen A. Functional role of T-cell receptor nanoclusters in signal initiation and antigen discrimination. Proc Natl Acad Sci U S A. (2016) 113:E5454–63. 10.1073/pnas.1607436113 27573839 PMC5027455

[9] Goyette J Nieves DJ Ma YQ Gaus K. How does T cell receptor clustering impact on signal transduction? J Cell Sci. (2019) 132:10. 10.1242/jcs.226423 30745330

[10] Lillemeier BF Mortelmaier MA Forstner MB Huppa JB Groves JT Davis MM. TCR and Lat are expressed on separate protein islands on T cell membranes and concatenate during activation. Nat Immunol. (2010) 11:90–6. 10.1038/ni.1832 20010844 PMC3273422

[11] Williamson DJ Owen DM Rossy J Magenau A Wehrmann M Gooding JJ. Pre-existing clusters of the adaptor Lat do not participate in early T cell signaling events. Nat Immunol. (2011) 12:655–62. 10.1038/ni.2049 21642986

[12] Rossy J Owen DM Williamson DJ Yang ZM Gaus K. Conformational states of the kinase Lck regulate clustering in early T cell signaling. Nat Immunol. (2013) 14:82–9. 10.1038/ni.2488 23202272

[13] Sherman E Barr V Manley S Patterson G Balagopalan L Akpan I. Functional nanoscale organization of signaling molecules downstream of the T cell antigen receptor. Immunity. (2011) 35:705–20. 10.1016/j.immuni.2011.10.004 22055681 PMC3225724

[14] Neve-Oz Y Razvag Y Sajman J Sherman E. Mechanisms of localized activation of the T cell antigen receptor inside clusters. Biochim Biophys Acta Mol Cell Res. (2015) 1853:810–21. 10.1016/j.bbamcr.2014.09.025 25300584

[15] Lee J Sengupta P Brzostowski J Lippincott-Schwartz J Pierce SK. The nanoscale spatial organization of B-cell receptors on immunoglobulin M- and G-expressing human B-cells. Mol Biol Cell. (2017) 28:511–23. 10.1091/mbc.e16-06-0452 27974642 PMC5305258

[16] Nunez MF Wisser K Veatch SL. Synergistic factors control kinase-phosphatase organization in B-cells engaged with supported bilayers. Mol Biol Cell. (2020) 31:667–82. 10.1091/mbc.E19-09-0507 31877064 PMC7202075

[17] Pageon SV Cordoba SP Owen DM Rothery SM Oszmiana A Davis DM. Superresolution microscopy reveals nanometer-scale reorganization of inhibitory natural killer cell receptors upon activation of NKG2D. Sci Signal. (2013) 6:11. 10.1126/scisignal.2003947 23882121

[18] Tolar P Sohn HW Pierce SK. The initiation of antigen-induced B cell antigen receptor signaling viewed in living cells by fluorescence resonance energy transfer. Nat Immunol. (2005) 6:1168–76. 10.1038/ni1262 16200067

[19] Tolar P Hanna J Krueger PD Pierce SK. The constant region of the membrane immunoglobulin mediates B cell-receptor clustering and signaling in response to membrane antigens. Immunity. (2009) 30:44–55. 10.1016/j.immuni.2008.11.007 19135393 PMC2656684

[20] Fiala GJ Kaschek D Blumenthal B Reth M Timmer J Schamel WWA. Pre-clustering of the B cell antigen receptor demonstrated by mathematically extended electron microscopy. Front Immunol. (2013) 4:10. 10.3389/fimmu.2013.00427 24367367 PMC3854567

[21] Rossboth B Arnold AM Ta H Platzer R Kellner F Huppa JB. TCRs are randomly distributed on the plasma membrane of resting antigen-experienced T cells. Nat Immunol. (2018) 19:821. 10.1038/s41590-018-0162-7 30013143 PMC6071872

[22] Brameshuber M Kellner F Rossboth BK Ta H Alge K Sevcsik E. Monomeric TCRs drive T cell antigen recognition. Nat Immunol. (2018) 19:487. 10.1038/s41590-018-0092-4 29662172 PMC7612939

[23] Simons K Ikonen E. Functional rafts in cell membranes. Nature. (1997) 387:569–72. 10.1038/42408 9177342

[24] Beck-García K Beck-García E Bohler S Zorzin C Sezgin E Levental I. Nanoclusters of the resting T cell antigen receptor (TCR) localize to non-raft domains. Biochim Biophys Acta (BBA) Mol Cell Res. (2015) 1853:802–9. 10.1016/j.bbamcr.2014.12.017 25535948

[25] Zhang W Trible RP Samelson LE. LAT palmitoylation: its essential role in membrane microdomain targeting and tyrosine phosphorylation during T cell activation. Immunity. (1998) 9:239–46. 10.1016/S1074-7613(00)80606-8 9729044

[26] Harder T Kuhn M. Selective accumulation of raft-associated membrane protein lat in T cell receptor signaling assemblies. J Cell Biol. (2000) 151:199–208. 10.1083/jcb.151.2.199 11038169 PMC2192654

[27] Cheng PC Brown BK Song W Pierce SK. Translocation of the B cell antigen receptor into lipid rafts reveals a novel step in signaling. J Immunol. (2001) 166:3693–701. 10.4049/jimmunol.166.6.3693 11238609

[28] Lichtenberg D Goni FM Heerklotz H. Detergent-resistant membranes should not be identified with membrane rafts. Trends Biochem Sci. (2005) 30:430–6. 10.1016/j.tibs.2005.06.004 15996869

[29] Sezgin E Levental I Mayor S Eggeling C. The mystery of membrane organization: composition, regulation and roles of lipid rafts. Nat Rev Mol Cell Biol. (2017) 18:361–74. 10.1038/nrm.2017.16 28356571 PMC5500228

[30] Owen DM Rentero C Magenau A Abu-Siniyeh A Gaus K. Quantitative imaging of membrane lipid order in cells and organisms. Nat Protoc. (2011) 7:24–35. 10.1038/nprot.2011.419 22157973

[31] Dinic J Riehl A Adler J Parmryd I. The T cell receptor resides in ordered plasma membrane nanodomains that aggregate upon patching of the receptor. Sci Rep. (2015) 5:10082. 10.1038/srep10082 25955440 PMC5386217

[32] Gaus K Chklovskaia E Fazekas de St Groth B Jessup W Harder T. Condensation of the plasma membrane at the site of T lymphocyte activation. J Cell Biol. (2005) 171:121–31. 10.1083/jcb.200505047 16203859 PMC2171224

[33] Zech T Ejsing CS Gaus K de Wet B Shevchenko A Simons K. Accumulation of raft lipids in T-cell plasma membrane domains engaged in TCR signalling. EMBO J. (2009) 28:466–76. 10.1038/emboj.2009.6 19177148 PMC2657588

[34] Owen DM Oddos S Kumar S Davis DM Neil MAA French PMW. High plasma membrane lipid order imaged at the immunological synapse periphery in live T cells. Mol Membr Biol. (2010) 27:178–89. 10.3109/09687688.2010.495353 20540668 PMC3870023

[35] Rentero C Zech T Quinn CM Engelhardt K Williamson D Grewal T. Functional implications of plasma membrane condensation for T cell activation. PLoS ONE. (2008) 3:e2262. 10.1371/journal.pone.0002262 18509459 PMC2384009

[36] Kabouridis PS Janzen J Magee AL Ley SC. Cholesterol depletion disrupts lipid rafts and modulates the activity of multiple signaling pathways in T lymphocytes. Eur J Immunol. (2000) 30:954–63. 10.1002/1521-4141(200003)30:3<954::AID-IMMU954>3.0.CO;2-Y 10741414

[37] Swamy M Beck-Garcia K Beck-Garcia E Hartl FA Morath A Yousefi OS. A cholesterol-based allostery model of T cell receptor phosphorylation. Immunity. (2016) 44:1091–101. 10.1016/j.immuni.2016.04.011 27192576

[38] Wang F Beck-García K Zorzin C Schamel WWA Davis MM. Inhibition of T cell receptor signaling by cholesterol sulfate, a naturally occurring derivative of membrane cholesterol. Nat Immunol. (2016) 17:844–50. 10.1038/ni.3462 27213689 PMC4916016

[39] Ashdown GW Williamson DJ Soh GHM Day N Burn GL Owen DM. Membrane lipid order of sub-synaptic T cell vesicles correlates with their dynamics and function. Traffic. (2018) 19:29–35. 10.1111/tra.12532 28981993

[40] Balagopalan L Yi J Nguyen T McIntire KM Harned AS Narayan K. Plasma membrane LAT activation precedes vesicular recruitment defining two phases of early T-cell activation. Nat Commun. (2018) 9:17. 10.1038/s41467-018-04419-x 29789604 PMC5964120

[41] Rudd-Schmidt JA Hodel AW Noori T Lopez JA Cho H-J Verschoor S. Lipid order and charge protect killer T cells from accidental death. Nat Commun. (2019) 10:5396. 10.1038/s41467-019-13385-x 31776337 PMC6881447

[42] Fischer K Voelkl S Berger J Andreesen R Pomorski T Mackensen A. Antigen recognition induces phosphatidylserine exposure on the cell surface of human CD8+ T cells. Blood. (2006) 108:4094–101. 10.1182/blood-2006-03-011742 16912227

[43] Davis RE Ngo VN Lenz G Tolar P Young RM Romesser PB. Chronic active B-cell-receptor signalling in diffuse large B-cell lymphoma. Nature. (2010) 463:88–92. 10.1038/nature08638 20054396 PMC2845535

[44] Burn GL Svensson L Sanchez-Blanco C Saini M Cope AP. Why is PTPN22 a good candidate susceptibility gene for autoimmune disease? FEBS Lett. (2011) 585:3689–98. 10.1016/j.febslet.2011.04.032 21515266

[45] Burn GL Cornish GH Potrzebowska K Samuelsson M Griffie J Minoughan S. Superresolution imaging of the cytoplasmic phosphatase PTPN22 links integrin-mediated T cell adhesion with autoimmunity. Sci Signal. (2016) 9:ra99. 10.1126/scisignal.aaf2195 27703032

[46] Shannon MJ Pineau J Griffie J Aaron J Peel T Williamson DJ. Differential nanoscale organisation of LFA-1 modulates T-cell migration. J Cell Sci. (2019) 133:jcs232991. 10.1101/602326 31471459 PMC7614863

[47] Moulton VR Tsokos GC. Abnormalities of T cell signaling in systemic lupus erythematosus. Arthritis Res Ther. (2011) 13:207. 10.1186/ar3251 21457530 PMC3132009

[48] Ali Abdalla M Mostafa El Desouky S Sayed Ahmed A. Clinical significance of lipid profile in systemic lupus erythematosus patients: relation to disease activity and therapeutic potential of drugs. Egypt Rheumatol. (2017) 39:93–8. 10.1016/j.ejr.2016.08.004

[49] Krishnan S Nambiar MP Warke VG Fisher CU Mitchell J Delaney N. Alterations in lipid raft composition and dynamics contribute to abnormal T cell responses in systemic lupus erythematosus. J Immunol. (2004) 172:7821–31. 10.4049/jimmunol.172.12.7821 15187166

[50] Miguel L Owen DM Lim C Liebig C Evans J Magee AI. Primary human CD4^+^ T cells have diverse levels of membrane lipid order that correlate with their function. J Immunol. (2011) 186:3505–16. 10.4049/jimmunol.1002980 21307290

[51] McDonald G Deepak S Miguel L Hall CJ Isenberg DA Magee AI. Normalizing glycosphingolipids restores function in CD4+ T cells from lupus patients. J Clin Investig. (2014) 124:712–24. 10.1172/JCI69571 24463447 PMC3904606

[52] Waddington KE Robinson GA Adriani M Rubio-Cuesta B Chrifi-Alaoui E Andreone S. Activation of the liver X receptors alters CD4^+^ T cell membrane lipids and signalling through direct regulation of glycosphingolipid synthesis. bioRxiv [Preprint]. (2019):721050. 10.1101/721050

[53] Jury EC Isenberg DA Mauri C Ehrenstein MR. Atorvastatin restores lck expression and lipid raft-associated signaling in T cells from patients with systemic lupus erythematosus. J Immunol. (2006) 177:7416–22. 10.4049/jimmunol.177.10.7416 17082661

[54] Yang W Bai Y Xiong Y Zhang J Chen S Zheng X. Potentiating the antitumour response of CD8+ T cells by modulating cholesterol metabolism. Nature. (2016) 531:651–5. 10.1038/nature17412 26982734 PMC4851431

[55] Ono A Freed EO. Plasma membrane rafts play a critical role in HIV-1 assembly and release. Proc Natl Acad Sci U S A. (2001) 98:13925–30. 10.1073/pnas.241320298 11717449 PMC61143

[56] Wu W Shi X Xu C. Regulation of T cell signalling by membrane lipids. Nat Rev Immunol. (2016) 16:690–701. 10.1038/nri.2016.103 27721483

[57] Simons K Ehehalt R. Cholesterol, lipid rafts, and disease. J Clin Invest. (2002) 110:597–603. 10.1172/JCI0216390 12208858 PMC151114

[58] Kenworthy AK. Nanoclusters digitize Ras signalling. Nat Cell Biol. (2007) 9:875–7. 10.1038/ncb0807-875 17671455

[59] Roob E III Trendel N Rein Ten Wolde P Mugler A. Cooperative clustering digitizes biochemical signaling and enhances its fidelity. Biophys J. (2016) 110:1661–9. 10.1016/j.bpj.2016.02.031 27074690 PMC4833775

[60] Suhaj A Gowland D Bonini N Owen DM Lorenz CD. Laurdan and Di-4-ANEPPDHQ influence the properties of lipid membranes: a classical molecular dynamics and fluorescence study. J Phys Chem B. (2020) 124:11419–30. 10.1021/acs.jpcb.0c09496 33275430

[61] Danylchuk DI Moon S Xu K Klymchenko AS. Switchable solvatochromic probes for live-cell super-resolution imaging of plasma membrane organization. Angew Chem Int Ed Engl. (2019) 58:14920–4. 10.1002/anie.201907690 31392763

[62] Barbotin A Urbancic I Galiani S Eggeling C Booth M Sezgin E. z-STED imaging and spectroscopy to investigate nanoscale membrane structure and dynamics. Biophys J. (2020) 118:2448–57. 10.1016/j.bpj.2020.04.006 32359408 PMC7231928

[63] Danylchuk DI Sezgin E Chabert P Klymchenko AS. Redesigning solvatochromic probe laurdan for imaging lipid order selectively in cell plasma membranes. Anal Chem. (2020) 92:14798–805. 10.1021/acs.analchem.0c03559 33044816

[64] Annibale P Scarselli M Kodiyan A Radenovic A. Photoactivatable fluorescent protein mEos2 displays repeated photoactivation after a long-lived dark state in the red photoconverted form. J Phys Chem Lett. (2010) 1:1506–10. 10.1021/jz1003523

[65] Platzer R Rossboth BK Schneider MC Sevcsik E Baumgart F Stockinger H. Unscrambling fluorophore blinking for comprehensive cluster detection via photoactivated localization microscopy. Nat Commun. (2020) 11:4993. 10.1038/s41467-020-18726-9 33020470 PMC7536177

[66] Veerapathiran S Wohland T. The imaging FCS diffusion law in the presence of multiple diffusive modes. Methods. (2018) 140–141:140–50. 10.1016/j.ymeth.2017.11.016 29203404

[67] Ritter AT Asano Y Stinchcombe JC Dieckmann NMG Chen B-C Gawden-Bone C. Actin depletion initiates events leading to granule secretion at the immunological synapse. Immunity. (2015) 42:864–76. 10.1016/j.immuni.2015.04.013 25992860 PMC4448150

